# Hormone-sensitive lipase deficiency alters gene expression and cholesterol content of mouse testis

**DOI:** 10.1530/REP-16-0484

**Published:** 2016-12-05

**Authors:** Feng Wang, Zheng Chen, Xiaofang Ren, Ye Tian, Fucheng Wang, Chao Liu, Pengcheng Jin, Zongyue Li, Feixiong Zhang, Baochang Zhu

**Affiliations:** College of Life SciencesCapital Normal University, Beijing, China

## Abstract

Hormone-sensitive lipase-knockout (HSL−/−) mice exhibit azoospermia for unclear reasons. To explore the basis of sterility, we performed the following three experiments. First, HSL protein distribution in the testis was determined. Next, transcriptome analyses were performed on the testes of three experimental groups. Finally, the fatty acid and cholesterol levels in the testes with three different genotypes studied were determined. We found that the HSL protein was present from spermatocyte cells to mature sperm acrosomes in wild-type (HSL+/+) testes. Spermiogenesis ceased at the elongation phase of HSL−/− testes. Transcriptome analysis indicated that genes involved in lipid metabolism, cell membrane, reproduction and inflammation-related processes were disordered in HSL−/− testes. The cholesterol content was significantly higher in HSL−/− than that in HSL+/+ testis. Therefore, gene expression and cholesterol ester content differed in HSL−/− testes compared to other testes, which may explain the sterility of male HSL−/− mice.

## Introduction

In 2010, an estimated 48.5 million couples worldwide had been unable to have a child in the previous 5 years (Mascarenhas *et al.* 2012). The testis plays a vital role in ensuring fertility by producing sperm, which penetrates the oocyte during fertilization. The testis contains multiple cell types, including macrophages, peritubular cells, Leydig cells (LCs), Sertoli cells (SCs) and germ cells, which interact to control spermatogenesis (Majumdar & Bhattacharya 2013). Spermatogenesis is a complex process for transmitting genetic information via fertilization, and by extension, continuing a species. Mammalian spermatogenesis is classically divided into three phases. The first phase is the proliferative or mitotic phase, in which germ cells undergo a series of mitotic divisions, transiting from SSCs (spermatogonial stem cells) to differentiated spermatogonia. In the second phase (meiotic phase), the spermatocytes undergo two consecutive divisions to produce haploid spermatids (Griswold & Oatley 2013). The third and the final phase is spermiogenesis, which is divided into the Golgi phase, the acrosome phase, the tail formation phase and the maturation phase (Cross 1996). During the Golgi phase, spermatid DNA begins to compact, and histones are replaced by protamines. During the acrosome phase, the Golgi complex surrounds the condensed nucleus, becoming the acrosomal cap, and the round cells elongate. Next, the centrioles elongate to become the tails of sperms. During the maturation stage, germ cells eject most of their cytoplasm and become mature sperm (Matzuk & Lamb 2008). All the spermiogenesis phases are accompanied by extensive changes to the cell membrane.

Hormone-sensitive lipase (HSL) is a complex multifunctional enzyme involved in fatty acid metabolism (Lampidonis *et al*. 2011). It hydrolyzes triacylglycerols, diacylglycerols, monoacylglycerols, cholesterol esters, retinyl esters and other lipids in multiple tissues (Kraemer & Shen 2002). HSL is expressed in several non-adipose tissues, including the ovaries (Varlamov *et al*. 2013), adrenal glands (Holysz & Trzeciak 2015), heart (Jaffer *et al*. 2012), skeletal and smooth muscle (Jocken *et al*. 2013), placenta (Waterman *et al*. 1998) and testes. Deletion of HSL from the mouse genome leads to male infertility by interruption of spermatogenesis (Osuga *et al*. 2000). HSL−/− mice were found to accumulate diacylglycerols and cholesterol esters in the testis (Osuga *et al*. 2000). Casado found that all lipids—including glycolipids, phospholipids and cholesterol esters of HSL substrates—are strictly distributed in male germ cells (Casado *et al*. 2013). Therefore, HSL not only plays a role in the provision of energy by mobilization of fat but also participates in other physiological processes as a multifunctional lipase. HSL deficiency triggers male sterility via an unclear mechanism.

In an effort to understand the function of HSL, we performed three experiments using wild-type (HSL+/+), heterozygous (HSL+/−) and knockout (HSL−/−) mice. First, we used immunohistochemistry to evaluate HSL distribution in the seminiferous tubules. Next, we compared the transcriptomes of testes of all three mouse strains. Finally, we determined the cholesterol contents of all three types of testes. We found that HSL knockout changed the gene expression and cholesterol content of HSL−/− testes. Thus, changes in gene expression and disturbances to cholesterol metabolism in testes might be the mechanisms underlying infertility in male HSL−/− mice.

## Materials and methods

### Ethics statement

HSL−/− mice (genetically modified C57BL/6 mice) were obtained from Prof. Grant A Mitchell, University of Montréal, Canada (Wang *et al*. 2001). HSL (+/+) mice were C57BL/6 purchased from the Military Medical Academy of China. All mice were handled in accordance with the institutional animal care policies of the Capital Normal University for two months. Mice were maintained under a 12-h light and 12-h darkness cycle in a specific pathogen-free stage at the Central Animal Laboratory of the Capital Normal University. The Laboratory Animal Care and Use Committee of the Capital Normal University approved this study.

### Genotyping and breeding

HSL-targeted disruption has been reported previously (Wang *et al*. 2001). Truncated HSL lacked 498 N-terminal amino acids, including all of exon 1. The LacZ gene was inserted downstream of the promoter of Lipe. Genotyping was performed using DNA isolated from the mouse tail. Tail biopsies (3 mm) were digested in 200 μL 50 mM NaOH and heated for 1 h at 95°C. DNA lysis solution was added to 20 μL of 10 mol/L Tris–HCl buffer (pH 8.0). PCR was performed in a 25 μL reaction (KT201, Tiangen, Beijing) with 1 μL template using a Bio-Rad s1000 PCR instrument (Bio-Rad) for 28 cycles, at an annealing temperature of 54°C, with an elongation for 90 s at 72°C. Three HSL oligo primers (Supplementary Table 1, see section on supplementary data given at the end of this article), including one forward primer and two reverse primers, were used. HSL-forward and HSL-reverse resulted in the amplification of a ~850 bp fragment from wild-type HSL, and HSL-forward and LacZ amplified a ~1 kbp fragment of exogenous LacZ, which was knocked into HSL. Three primers were added to the PCR system synchronous for genotyping. PCR products were resolved by agarose gel electrophoresis. Genotypes were determined according to migration pattern (Supplementary Fig. 1). HSL−/− male pups were used to study infertility, whereas HSL+/− male and HSL−/− female pups were mated to provide further experimental animals.

### Immunohistochemistry

**Figure 1:**
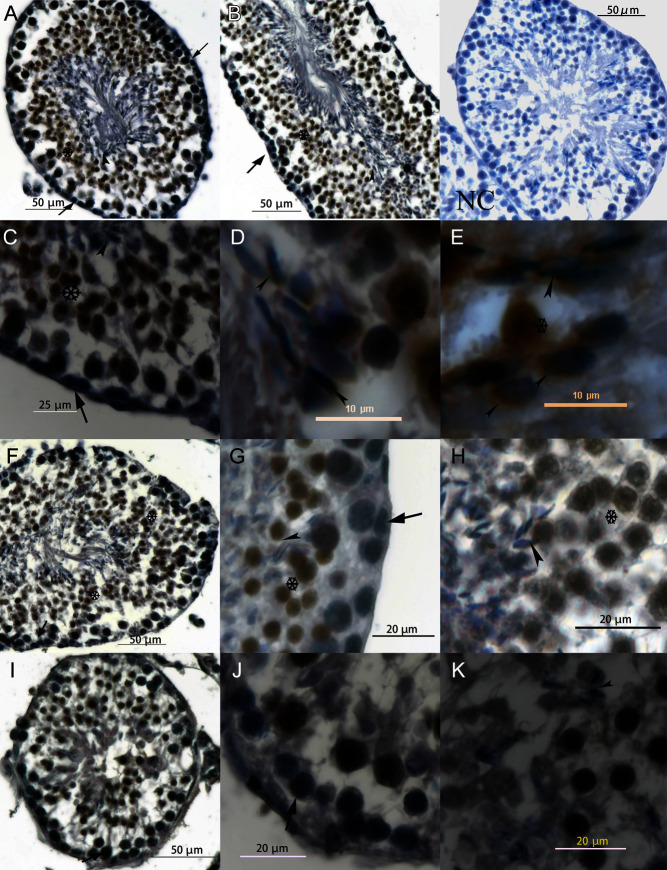
Localization of HSL in seminiferous tubules. A and B show transverse and longitudinal sections of HSL+/+ seminiferous tubules. C, D and E show the distribution of HSL within HSL+/+ sperm heads around the acrosome, which covers agglutinated sperm nucleus. F, G and H are HSL+/− testis. G and H show the basement membrane and lumen of seminiferous tubules. I, J and K show HSL−/− testis. J and K show the basement membrane and abluminal. NC is negative HSL+/+ testes control, in which all the steps excepting HSL antibody incubated were taken. And there is no obvious HSL-DAB noise signal around sperm head. Postmeiotic germ cells are more evident in HSL+/+ and HSL+/− testes than HSL−/− testes, and there was almost no forming sperm head in HSL−/− testes. Arrows indicate early germ cells, which did not express HSL protein. Arrowheads show the elongating spermatid, and DAB stained the pre-acrosome around HSL protein. Cells surrounding the asterisk exhibited a strong HSL-DAB signal, from primary spermatogonia to the mature sperm in the lumen of seminiferous tubules.

Testes were fixed in Bouin’s Fluid (HT10132, Sigma) overnight at 4°C. They were then dehydrated through a graded series of ethanol, cleared in dimethyl benzene and soaked with melting paraffin. Paraffin sections (8 μm thickness) were mounted on slides coated with polylysine (FSH0010; TDB-science, Tianjin, China) and dried overnight at 42°C. After dewaxing, rehydration and antigen retrieval with sodium citrate buffer, endogenous peroxidase activity was inhibited by the addition of H_2_O_2_. Nonspecific binding was blocked by incubation with 10% goat serum (Zhongshan Golden Bridge Biotech Co., Beijing, China) in PBS for 1 h. Sections were incubated with a rabbit anti-mouse HSL polyclonal immunoglobulin G (IgG) antibody (H-300; Santa Cruz Biotechnology), specific for the region containing amino acids 476–775 mapping at the C-terminus of HSL, in 10% goat serum overnight at 4°C. Sections were incubated with goat anti-rabbit IgG, followed by incubation with secondary antibodies linked to HRP. The DAB detection system (detecting S-A/HRP-DAB; Zhongshan Golden Bridge Biotech Co.) was used. Addition of the DAB colorant to S-A/HRP resulted in the generation of an orange color. As a negative control, the antibody was replaced with a control rabbit IgG. Sections were counterstained with Masson’s hematoxylin (Shanghai Chemical Reagent Co., Shanghai, China). Negative HSL+/+ testes control (NC in Fig. 1) avoided nonspecific binding. All steps were undertaken except HSL antibody incubation in NC group. In order to confirm the HSL knockout mice still expressed truncated HSL, we analyzed *Hsl* mRNA expression based on transcriptome data as per description followed (Supplementary Fig. 2A) and peptide (Supplementary Fig. 2B) in testes from each mice genotype. Anti-HSL antibody revealed two bands around 130 kDa, indicating that there may be isomers in the testes of wild-type mice. Sections were examined using a Nikon NI microscope (Nikon).

### Protein extraction and western blot

Testes were collected, and western blotting was performed as described previously (Ding *et al*. 2016). The samples were homogenized in a buffer containing Tris (hydroxymethyl) aminomethane hydrochloride (20 mM Tris–HCl, pH 7.5), sucrose (0.25 M), MgCl_2_ (2.5 mM), EDTA (2.5 mM), KCl (10 mM), thimerosal (0.02%) and a protease inhibitor cocktail (Sigma-Aldrich). The homogenate was centrifuged for 10 min (14,000 ***g***, 4°C, 30 min). All protein extractions were carried out on ice. Protein concentrations in each sample were determined using the Bradford assay (Bio-Rad Laboratories). Cell lysate aliquots containing 15 μg protein were subjected to SDS-PAGE (10%) and then electrophoretically transferred to a nitrocellulose membrane for 2 h. After transfer, the membranes were blocked with 5% (w/v) bovine serum albumin (BSA) in TBST (20 mM Tris–HCl, 150 mM NaCl, 0.05% Tween 20, pH 7.5) buffer for 1 h at room temperature. Antibodies against HSL (1:5000) and against β-actin (1:2500) were incubated (4°C, overnight) with the membranes, which were subsequently treated with a secondary antibody, goat anti-rabbit IgG conjugated to horseradish peroxidase (HRP) (1:2500) in blocking solution for 1 h at room temperature. After being washed three times with TBST buffer, immunoreactive bands were visualized with the SuperSignal West Pico Chemiluminescent Substrate (Thermo Fisher Scientific) according to the manufacturer’s instructions, and the protein content was determined by densitometrically scanning the exposed bands with ImageQuant LAS 4000 (GE Healthcare Life Sciences). Immunoreactive signals were analyzed using gel-pro Analyzer 4.0.

### Transcriptome analysis of mouse testis

Testes collected from mice were frozen in liquid nitrogen immediately and stored at −80°C prior to total RNA extraction. Transcriptome analyses were performed on total mRNA extracted from the testes. RNA was isolated from testes using TRIzol (Invitrogen) and used to generate cDNA templates. Total RNA integrity was detected using denatured agarose gel and an Agilent 2100 Bio-analyzer (Agilent Technologies), and the concentration and purity were detected by NanoDrop 2000 (NanoDrop, Wilmington, DE, USA). Samples with an RNA integrity number (RIN) of ≥7.5 and OD260/280 ratio of 1.8–2.0 were subjected to RNA-seq.

We constructed three cDNA libraries of the three genotypes (Supplementary Table 2) and performed transcriptome sequencing. Oligo (dT) magnetic beads were used to separate mRNA from total RNA. Three cDNA libraries were prepared using a TruSeq RNA sample preparation kit from Illumina (San Diego, CA, USA) and random primers. After second-strand cDNA synthesis, fragments were end-repaired, and a poly-A tail was added and ligated with indexed adapters. The products were purified and enriched using 15 PCR cycles to generate the final cDNA library. The tagged cDNA libraries were pooled in equal ratios and used for 151 bp paired-end sequencing in a single lane of an Illumina HiSeq4000 instrument (base composition map is shown in Supplementary Fig. 3).

**Figure 2:**
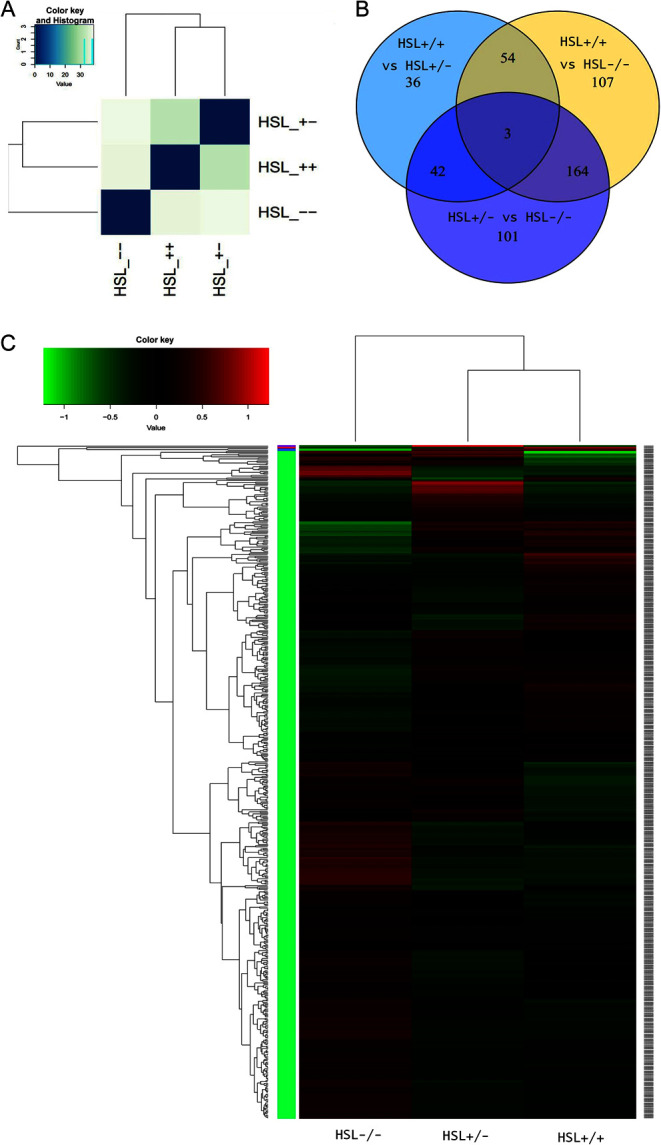
Analysis of differences among the groups. (A) Entire genetic correlation analysis among HSL−/−, HSL+/+ and HSL+/−. The similarity between groups meets experimental design expectations. The similarity between HSL+/+ and HSL+/− is greater than HSL−/−. (B) Venn diagram of DEGs of each genotype group, created using Venn diagram software. Different numbers indicate DEGs between groups. Circles represented with different colors were compared between the three genotypes. Definition of differential expression genes by Cufflinks, *P* < 0.01. (C) Cluster analysis between samples. Each column represents a genotyping sample. Each row represents a gene. Red indicates higher expression, and green indicates lower expression. A gene-clustering tree is shown on the left with h-cluster clustering. A sample-clustering tree is shown at the top. As the distance between branches decreases, the similarity in expression increases.

The RNA-seq reads were initially processed to remove the adapter and primer sequences using the SeqPrep and Sickle software. We collected 6 Gb of raw data, 5.5 Gb of clean data and 40 Mb of clean reads (Supplementary Table 2). The high-quality reads (Supplementary Figs 3 and 4) were aligned to the reference genome using TopHat (Trapnell *et al*. 2012). The unit of measurement used by Cufflinks to estimate transcript abundance was fragments per kilobase of exon per million fragments mapped (FPKM scores; shown in Supplementary Fig. 4) (Trapnell *et al*. 2013). The genes of three testis transcriptomes (24,718, 24,516 and 24,648 genes were expressed in HSL+/+, HSL+/− and HSL−/− testes respectively) were compared to those present in the Ensembl database (Fig. 2C). Over 50% of all known mouse genes (41,386 genes in Ensembl database) were expressed in the testes. Differentially expressed genes (DEGs) were analyzed using the Cuffdiff module of the Cufflinks package. The sequences were further annotated using the GO and KEGG databases. We categorized gene function with an emphasis on biochemical pathways. Goatools was used for GO enrichment analysis. KOBAS was used for KEGG pathway enrichment analysis (Xie *et al*. 2011) (example is shown in Supplementary Fig. 5).

### Quantitative real-time PCR analysis

RNA was extracted as transcriptome sample preparation steps. RNA (1 μg) was reverse transcribed into cDNA using a FastQuant RT Kit with gDNase (KR106; Tiangen, Beijing, China) according to the manufacturer’s protocol. Fluorescence-based quantitative real-time PCR (qRT-PCR) was performed using SYBR-Green Mix (Transgene, Beijing, China) according to the manufacturer’s protocol. Amplification data were collected using the Techne pro software (Techne, Bibby Scientific, Stone, UK) and the Techne Prime Pro 48 instrument (Techne, Bibby Scientific). Oligonucleotide primers specific to each target gene were designed (Supplementary Table 1). The 2^−ΔΔCT^ method was used for the quantification of relative changes in gene expression and normalized to Gapdh. . Expression in wild-type testes was used as the control. PCR amplification was performed in 40 cycles comprising denaturation at 95 °C for 15 s, annealing at 55°C for 15 s and extension at 72 °C for 30 s. A melt curve was then generated from 55°C to 95°C (Supplementary Fig. 6). Furthermore, qRT-PCR was employed to check the reliability and reproducibility of the DEGs. Values are means of three mice.

### Gas chromatography–mass spectrometry fatty acid analysis

Four testes samples from each genotype group were cryopreserved at −80°C overnight. Testes were dried using FreeZone Freeze Dry Systems (Labconco, Kansas City, KS, USA), and their dry weight was recorded. Standards (EC10A-1KT, CRM1891) were obtained from Sigma Chemical. The gas chromatographic system comprised a DB-23 detector (60.0 m × 250 µm × 0.25 µm) interfaced with an Agilent HP-6890 gas chromatograph. The oven temperature was initially held at 60°C for 2 min, and then increased to 260°C at a rate of 3°C/min. The inlet temperature was 270°C for 8 min. Split injection was conducted with a split ratio of 1:30, and helium carrier gas was used at a constant flow rate of 2.0 mL/min. Gas chromatography–mass spectrometry (GC/MS) was performed using 70 eV electron ionization. Peak identification was achieved by comparison with known standards. FAME concentrations were estimated using calibration curves constructed with the external standards at six concentrations according to the internal standard method (Seifi *et al*. 2014). All procedures described previously conformed with standards GB/T 21514-2008/ISO/TS 17764:2002 (determination of the content of fatty acids in feeds).

### Testicular cholesterol concentration

Testes from HSL (+/+), HSL (+/−) and HSL (−/−) mice were cryopreserved at −80°C overnight, and then dried using FreeZone Freeze Dry Systems (Labconco). Testes dry weights were recorded. Extraction was performed using 3 mL ethyl alcohol. The solution was centrifuged and 2 mL of the absorbed supernatant liquid was used as the total cholesterol solution to be stained. Total cholesterol (free cholesterol and cholesteryl ester) was assayed using ferric chloride–phosphoric and sulfuric acid (S–P–Fe) according to a protocol reported previously (Rossouw *et al*. 1976), and solution was detected by a microplate reader (Synergy HT; Biotek). To produce the Fe color agent, FeCl_3_·6H_2_O (0.2 g) was added to 8 mL phosphate solution and dissolved in 100 mL concentrated sulfuric acid. Cholesterol was diluted by the gradient of 8 μg/mL as a cholesterol standard solution to generate the standard curve (Supplementary Fig. 7). Two milliliters of cholesterol solution were added to 2 mL S–P–Fe color agent, mixed and 200 μL was transferred to wells of a 96-well plate. Absorbance at a wavelength of 550 nm was then determined using a microplate reader (Synergy HT; Biotek Co.). Pure ethyl alcohol was used as the negative control.

## Results

### HSL distribution in seminiferous tubules

We measured the body weights of all mice daily between days 21 and 45. The three groups of male and female mice grew similarly. HSL−/− and HSL+/− mice were of normal body weight (Supplementary Fig. 8). We cannot find the differences in growth on each genotype. We explored the HSL protein distribution in HSL+/+, HSL+/− and HSL−/− testes using a rabbit anti-mouse HSL polyclonal antibody (Fig. 1). HSL was expressed by all the spermatocyte cells to mature sperm acrosomes in HSL+/+ testes (Fig. 1A, B, C, D and E). The first exon of HSL was replaced by exogenous LacZ, but the C-terminal region, where the ligand for anti-HSL antibody was located, was present in the mRNA and protein (Supplementary Fig. 2). Therefore, the truncated protein of HSL−/− testes was immunohistochemically stained in all cells, from spermatocyte cells to pro-apoptotic germ cells. We found that fewer cells were stained in HSL−/− than those in HSL+/+ and HSL+/− testes.

### Transcriptome analysis of testes of HSL−/− mice

**Figure 3:**
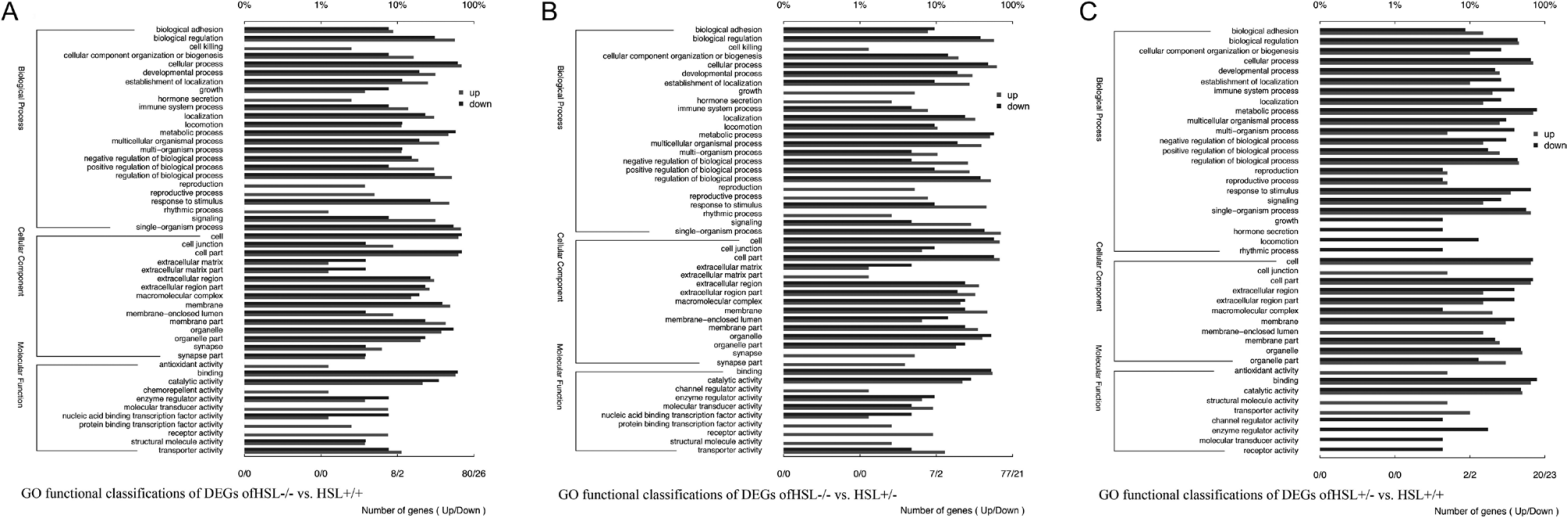
GO functional classifications of DEGs between HSL−/−, HSL+/− and HSL+/+ testes transcriptome. (A) GO functional classifications of DEGs of HSL−/− vs HSL+/+. (B) GO functional classifications of DEGs of HSL−/− vs HSL+/−. (C) GO functional classifications of DEGs of HSL+/− vs HSL+/+. The bottom horizontal axis shows the number of genes and GO term. The top horizontal axis shows the proportion of the number of GO processes to the total number of DEGs. The vertical axis shows alteration of each GO term. The three partitioned areas represent the biological process, cellular component and molecular function GO subcategories. Red and blue bars indicate upregulated and downregulated genes, respectively. Significance analysis by GoTools, *P* < 0.05.

**Figure 4:**
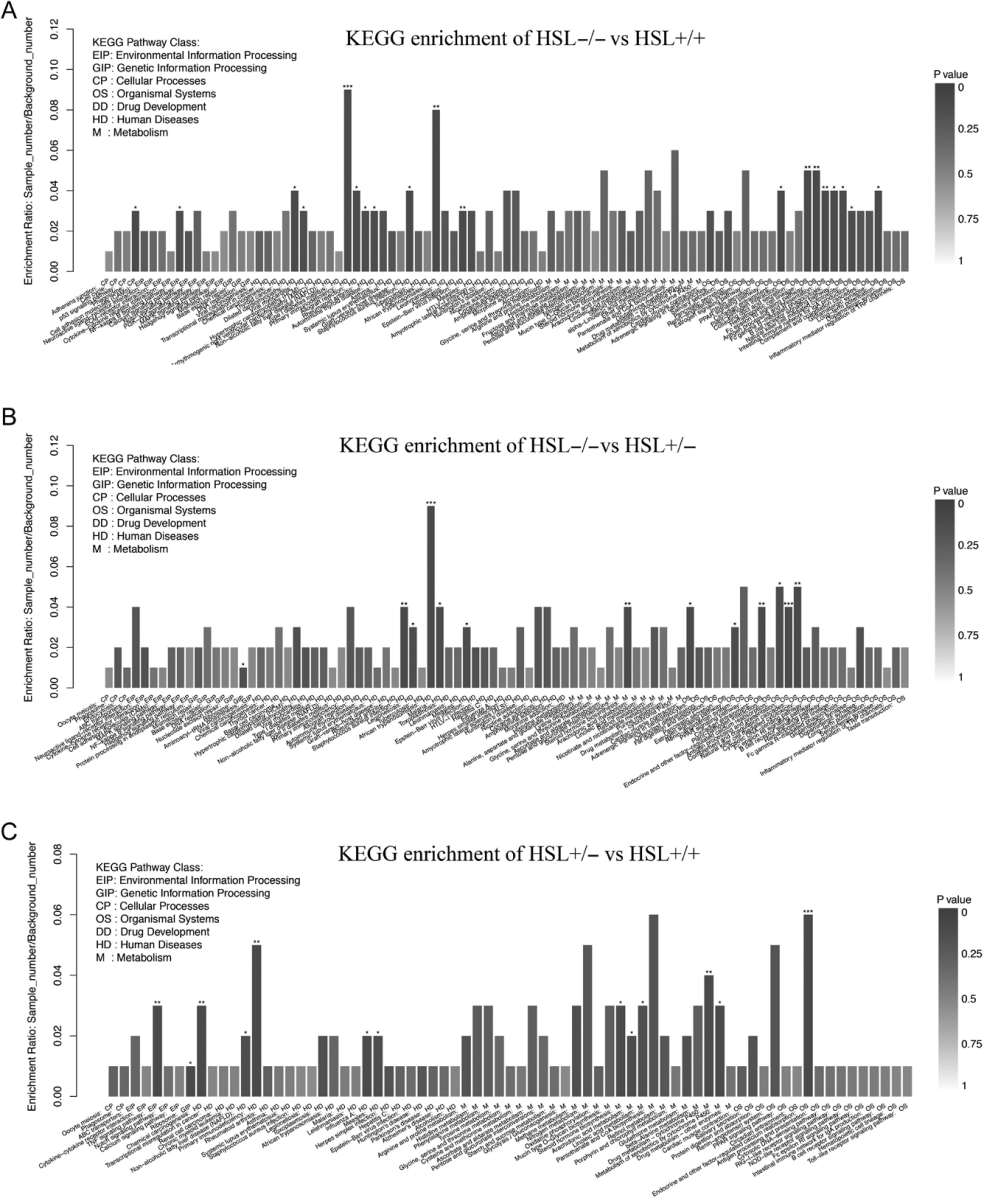
KEGG enrichment of DEGs among HSL−/−, HSL+/− and HSL+/+ testes. The transcripts were matched with the KEGG database. KEGG enrichment of DEGs between (A) HSL−/− and HSL+/+, (B) HSL−/− and HSL+/− and (C) HSL−/+ and HSL+/+. Each column is a pathway. The horizontal axis shows the process name and classification, color indicates statistical significance, and the color intensity indicates the degree of significance. Significance analysis by KOBAS. *P* < 0.01; ***P < 0.001; **P < 0.01; *P < 0.05.

In accordance with the overall profile (Fig. 2A), the transcriptome of HSL−/− testes differed significantly from those of the other two groups. The expression of 328, 310 and 135 genes differed significantly between HSL−/− and HSL+/+ mice, HSL−/− and HSL+/− mice and HSL+/− and HSL+/+ mice respectively (Fig. 2B). A total of 167 genes differed significantly in terms of expression between HSL−/− mice and the other two groups. GO analyses were performed to identify functions differing between HSL−/− and HSL+/+ (Fig. 3A), HSL−/− and HSL+/− (Fig. 3B) and HSL+/− and HSL+/+ (Fig. 3C). The three principal categories evident upon GO functional annotation were biological process (BP, GO: 0008150), cellular component (CC, GO: 0005575) and molecular function (MF, GO: 0003674). KEGG pathway analysis (Fig. 4) showed that DEGs were involved in cell death, the inflammatory response, lipid metabolism, steroidogenesis and steroid hormone synthesis (example is shown in Supplementary Fig. 5), cell junction and maintenance in testes and the reproductive process. Compared to the control (HSL+/+), 200 DEGs defined by 50 GO terms were upregulated in HSL−/− testes, including 11 molecular function pathways, 15 cellular component pathways and 24 biological processes. The 121 downregulated genes bore 40 GO terms, of which 6 were molecular function pathways, 15 were cellular component pathways and 19 were biological process pathways. A comparison of the DEGs of HSL−/− vs HSL+/− (Fig. 2B) testes yielded data similar to those afforded upon comparison of HSL−/− vs HSL+/+ testes. The expression level of 310 genes (200 upregulated and 110 downregulated) differed significantly between HSL−/− and HSL+/− testes. Most of these genes bore 49 GO terms, with the exception of chemorepellent activity, the extent of which was similar between the DEGs of HSL−/− and HSL+/+ testes. There was a minimal difference in the expression profile between HSL+/− and HSL+/+ testes (Fig. 2C). Such genes were involved in 43 GO terms, but only 135 genes (71 upregulated and 64 downregulated) differed significantly in terms of expression levels. These DEGs do not lead to male infertility.

A total of 157, 145 and 106 differences in annotated KEGG pathways were identified when HSL−/− and HSL+/+ (Fig. 4A), HSL−/− and HSL+/− (Fig. 4B) and HSL+/− and HSL+/+ testes (Fig. 4C) respectively, were compared. KEGG enrichment analysis suggested that endocrine or signal transduction pathways, the estrogen signaling pathway and steroid hormone biosynthesis were significantly enriched. KEGG annotation also indicated that certain genes that are involved in cholesterol and fatty acid metabolism, glycol metabolism, phagocytosis, P450-related functions and cell adhesion were deregulated.

**Figure 5:**
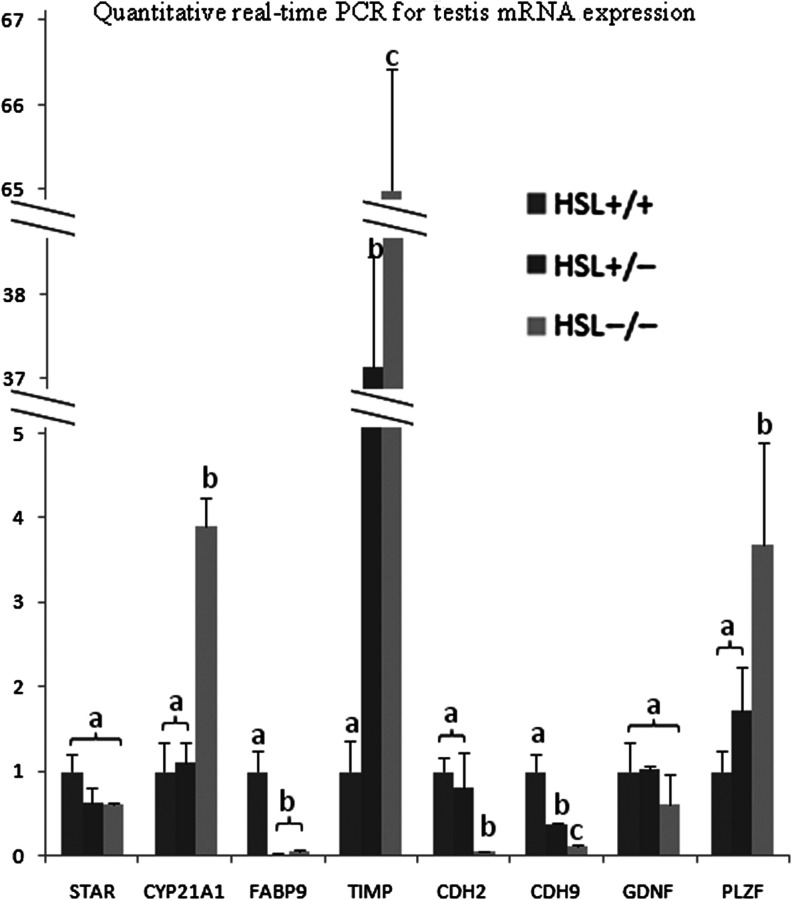
Quantitative real-time PCR validation of the sequencing data. STAR, CYP21A1 and FABP9 are associated with fat metabolism. TIMP, CDN2 and CDH9 are related to intercellular adhesion. GDNF and PLZF participate in stem cell self-renewal. The trends in the expression of most genes (except PLZF) were similar to those determined by sequencing. There is no significant difference expression of PLZF between each genotypes by transcriptome. Error bars indicate s.d. a, b and c indicate significant differences. Groups are statistically different by one-way ANOVA (*P* < 0.05).

To validate the sequencing data, eight genes were selected for qRT-PCR analysis (Fig. 5). STAR, CYP21A1 and FABP9 are associated with fat metabolism; TIMP, CDH2 and CDH9 play roles in intercellular adhesion; and GDNF and PLZF participate in stem cell self-renewal. The expression trends of most of these genes (with the exception of PLZF) were similar to those indicated by sequencing. PLZF, encoded by Zbtb16, prevents spermatogonial differentiation (Filipponi *et al*. 2007). However, experimental methodology (Nickells & Pelzel 2015) and PLZF expression differed when the sequencing and qRT-PCR data were compared. The sequencing data showed no significant effect in all three testes; however, qRT-PCR data showed a significant effect in HSL−/− testis. Expression of GDNF, which maintains SSC self-renewal in early germ cells, did not differ significantly among the three testes when evaluated using RNA-seq or qRT-PCR.

### Total fatty acid and cholesterol contents in the testes of HSL−/− mice

Testicular cells are richer in polyunsaturated fatty acids (PUFA) than the cells of other organs. HSL hydrolyzes glycerides of the lipid droplets that are in the cytoplasm of the cells (Welte 2015). We hypothesized that the deregulation of fatty acid concentrations in HSL−/− testis blocks spermatogenesis. Total fatty acid concentrations were determined by GC/MS, which indicated no significant differences in the levels of the fatty acids of interest between HSL−/− and HSL+/− or HSL+/+ testis (Table 1). Therefore, HSL must exert effects other than fatty acid mobilization in the testis.

**Table 1:** Fatty acid composition of mice testes. Lipid fatty acid levels are expressed as mean percentages ± s.d. to the total fatty acid levels. There was no significant difference among the groups by chi-squared test by SPSS22.0.

**FFA**	**HSL+/+** (%)	**HSL+/−**(%)	**HSL−/−** (%)
C6:0	0.066 ± 0.024	0.065 ± 0.063	0.090 ± 0.035
C8:0	1.303 ± 0.367	1.358 ± 0.008	1.532 ± 0.475
C10:0	0.044 ± 0.005	0.053 ± 0.008	0.055 ± 0.005
C12:0	0.095 ± 0.048	0.062 ± 0.161	0.083 ± 0.028
C14:0	0.902 ± 0.105	0.71 ± 0.074	0.789 ± 0.031
C14:1	0.050 ± 0.005	0.052 ± 0.004	0.058 ± 0.006
C15:0	0.171 ± 0.015	0.198 ± 0.016	0.159 ± 0.014
C16:0	27.718 ± 1.677	26.185 ± 1.139	26.273 ± 3.226
C16:1	2.919 ± 0.714	3.185 ± 0.253	3.354 ± 0.921
C17:0	0.319 ± 0.102	0.289 ± 0.044	0.283 ± 0.059
C18:0	5.729 ± 0.423	5.154 ± 1.281	5.688 ± 0.701
C18:1n9c	23.964 ± 0.657	23.540 ± 1.029	23.796 ± 1.347
C18:2n6c	22.610 ± 1.565	22.778 ± 1.018	22.221 ± 3.946
C18:3n3	1.026 ± 0.129	1.079 ± 0.267	0.915 ± 0.105
C20:0	0.101 ± 0.018	0.133 ± 0.036	0.109 ± 0.022
C20:1	0.607 ± 0.066	0.687 ± 0.072	0.611 ± 0.098
C21:0	0.469 ± 0.032	0.497 ± 0.043	0.494 ± 0.037
C20:3n6	0.675 ± 0.093	1.086 ± 0.123	0.777 ± 0.087
C20:4n6	5.771 ± 0.724	6.848 ± 0.363	6.651 ± 0.882
C20:5n3	0.133 ± 0.041	0.12 ± 0.006	0.149 ± 0.016
C22:0	0.100 ± 0.003	0.237 ± 0.009	0.127 ± 0.009
C22:1n9	0.048 ± 0.006	0.105 ± 0.018	0.050 ± 0.009
C23:0	0.081 ± 0.020	0.108 ± 0.010	0.084 ± 0.029
C24:0	0.358 ± 0.101	0.602 ± 0.056	0.445 ± 0.025
C22:6n3	4.282 ± 0.521	4.712 ± 0.566	4.696 ± 0.829
C24:1	0.202 ± 0.035	0.518 ± 0.061	0.235 ± 0.025

**Figure 6:**
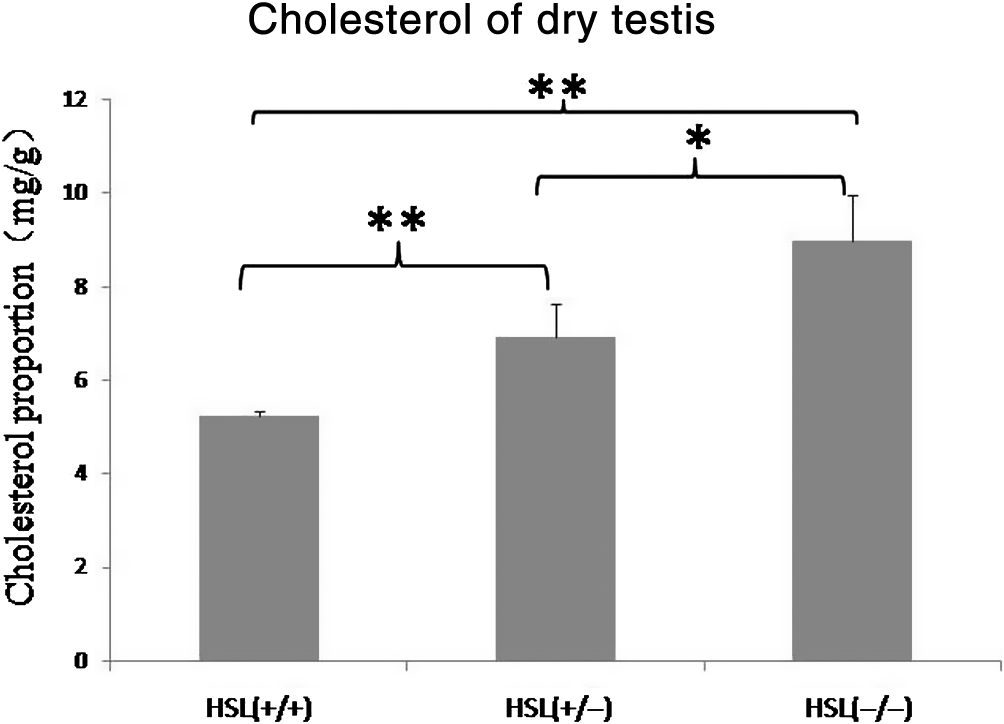
Cholesterol concentration in dry testes. Cholesterol concentrations differ significantly among HSL−/−, HSL+/− and HSL+/+ mouse testes. HSL−/− testis exhibited the highest cholesterol concentration, which may have blocked spermatogenesis. Values are means of four biological replicates; error bars indicate s.d. Means were compared by one-way ANOVA following Tukey *post hoc* test by SPSS 22.0. *P < 0.05, **P < 0.01.

The cholesterol content of dry HSL−/− testes was significantly higher than that of HSL+/− and HSL+/+ (Fig. 6). Cholesterol concentration increased as HSL expression decreased. Our data suggest that HSL was a principal cholesterol ester hydrolase in the testis, in line with the results of previous study (Hermo *et al*. 2008) because HSL knockout led to the accumulation of cholesterol esters.

## Discussion

HSL hydrolyzes triacylglycerols, diacylglycerols, monoacylglycerols, cholesterol esters, retinyl esters and other lipids, thus playing various roles in fatty acid metabolism (Kraemer & Shen 2002). It has been reported that HSL mediates the variation in fat content that is evident in obese patients (Wang & Huang 2012, Varlamov *et al*. 2013). However, we found that both male and female HSL−/− mice exhibited normal growth curves and were not obese (Supplementary Fig. 8), and similar to the data of a previous report (Chung *et al*. 2001), HSL deactivation does not lead to severe lipid metabolism-related disease. Adipose tissue contains another lipase–adipose triglyceride lipase (ATGL) (Zimmermann *et al*. 2004) that regulates fatty acid metabolism. ATGL and HSL share similar catalytic activities. However, HSL supports specific physiological functions such as spermatogenesis and tissue development (example: that of nerve tissue) (Osuga *et al*. 2000, Haemmerle *et al*. 2002).

HSL is a critical enzyme involved in spermatogenesis, as revealed by the infertility of male HSL−/− mice (Osuga *et al*. 2000, Chung *et al*. 2001); however, the precise role played by this enzyme remains unclear. We hypothesized that disruption of glyceride and cholesterol ester hydrolysis might lead to the accumulation of substrates or the lack of products required for germ cell elongation. Whether HSL knockout leads to damage to somatic cells or germ cells is unclear. LCs secrete a variety of factors that influence the SSC niche. SCs not only secrete factors for germ cells but also form the blood–testis barrier (BTB) by interacting with the basement membrane to produce a niche microenvironment suitable for SSCs (negative colored in Fig. 1). Both cell types are essential for spermatogenesis. A high level of expression of HSL was detected in elongated spermatids in human and rodent seminiferous tubules (Blaise *et al*. 2001). We assessed the distribution of HSL protein in the testes of HSL+/+, HSL+/− and HSL−/− mice. HSL was expressed from the spermatogonial cells that had left the niche to mature sperm acrosomes (cells surrounding asterisk in Fig. 1) in the adluminal compartment. We found that truncated HSL was expressed from spermatogonial cells to pre-rupture germ cells, in the testes of HSL−/− mice (Fig. 1I, J and K). In the absence of HSL, spermatogenesis was blocked at the stage of germ cell elongation after meiosis; thus, spermiogenesis was incomplete (Fig. 1J), and no mature sperm were evident in HSL−/− testes (Fig. 1K). The HSL expression diagram and western blot (Supplementary Fig. 2) indicate that the target of the HSL antibody (the C-terminal) was present, despite the replacement of the N-terminal with LacZ. The distribution of HSL suggests that the enzyme is important in terms of spermiogenesis. Therefore, an important pathway of lipid metabolism must be lost in HSL−/− testes causing cell differentiation to be suspended. Considering the features of HSL reported by early studies (Anderson *et al*. 2002), the deregulated ester must be a lipid such as a glycerol or cholesterol ester. More research is required to reveal the detailed cause of infertility in HSL−/− male mice.

HSL knockout blocks the blocking of spermatogenesis, which may, in turn, change the gene expression levels of other genes. It was important to compare the DEGs of HSL−/− to those of the other strains in terms of lipid metabolism, reproductive progression and HSL-related biological processes. The transcriptome data identified the genes involved in testis-specific biological processes (Musiolik 1971, Lee *et al*. 2012, Waclawska & Kurpisz 2012). HSL knockout affected the expression levels of many genes of lipid metabolism because HSL hydrolyzes glycerol and cholesterol esters. The transcriptome results showed that 37 lipid metabolism genes were upregulated and 15 were downregulated. The downregulated genes Atp6v0e2 (ATPase, H^+^-transporting, lysosomal V0 subunit E2), Tgtp2 (T cell-specific GTPase 2) and Galntl6 are involved in energy metabolism and glucose metabolism respectively. Downregulation of these three genes in HSL−/− testes will affect the energy metabolism. We found that HSL inactivation reduced energy metabolism in the testis. The four-fold upregulated gene (Fabp4, fatty acid-binding protein 4) encodes an intracellular protein participating in the metabolism of long-chain fatty acids, retinoids and other hydrophobic ligands (Liu *et al*. 2008). Three dysregulated cytochrome P450 subfamily members, Cyp4v3, Cyp2e1 and Cyp21a1, promote the oxidation of cholesterol to testosterone by LCs (Zhou *et al*. 2015). PPARα, which is involved in fatty acid metabolism, was upregulated more than three-fold to enhance the energy metabolism of SCs and germ cells (Regueira *et al*. 2014). G-protein-coupled receptor C3aR1, which is located in the membrane, was five-fold upregulated in HSL−/− testes. C3aR1 mediates the functions of insulin by modulating insulin resistance, obesity and macrophage function (Hannedouche *et al*. 2013). We found that the immunoglobulin-like domain containing receptor 2 (Ildr2) was significantly upregulated. A previous report found that an Ildr2-knockdown mouse accumulated lipids in the liver (Nakata *et al*. 2012). Haptoglobin (Hp) is expressed in SCs, LCs and germ cells, but not in the epididymis (O’Bryan *et al*. 1997). It plays important roles in both iron metabolism and inflammation in the testis (Gloria-Bottini *et al*. 2009). Lipids are a major source of energy, and energy metabolism will be affected by a change in the lipid profile of HSL−/− testes.

Cell membranes are lipid bilayers. Membrane remodeling is thus entwined with lipid metabolism (Chap 2016). HSL−/− mice testes were lacking some lipid hydrolysis reactions. Several membrane formation/location pathways were found to be dysregulated upon transcriptome analysis. Receptor activity-modifying protein 1 (RAMP1), which facilitates the localization of receptors on the cell surface (Li *et al*. 2014), was increased five-fold in HSL−/− testes. Myelin protein zero-like 2 (Mpzl2), a cell adhesion molecule of the immunoglobulin superfamily, is present on the surface of early spermatogonial cells (Nakata *et al*. 2012) and was upregulated in HSL−/− testes. Abnormal membrane components, such as CDH2 and CDH9, trigger changes in intercellular connections and dissociate adherent cells. Membrane remodeling during spermatogenesis plays a critical role in the formation of the sperm head. Once compromised, spermatogenesis is paused in the most intense period of deformation of HSL−/− mice. As late germ cells are lacking in HSL−/− testes, germ cell differentiation in such testes will be compromised. Four DEGs of the reproductive process (Hp, Timp1, Sptbn4 and P2ry2) were expressed in the HSL−/− testis. It is likely that all genes expressed in the testis participate directly or indirectly in male reproductive processes. Several genes involved in the inflammatory processes were dysregulated in HSL−/− testes. Spermiogenesis ceased during elongation to spermatozoons. The expression levels of many genes are influenced by the inflammation processes; such genes include B2m, Pde7b, Ighg1 and Igha. Most are expressed in immune and inflammatory cells. Such genes might induce repair of testis tissue. Interestingly, the genes upregulated in HSL−/− vs HSL+/+ mouse testes were defined by only 10 GO terms. Such genes upregulated upon HSL inactivation, may be involved in the induction of spermatogenesis failure, or the failure to self-repair spermatogenesis. Of all 41,386 DEGs, the levels of 328 (0.7%) were altered in HSL−/− testes of infertile male mice, but changes in transcription levels were modest. Germ cells underwent normal stem cell differentiation and proliferation to generate pre-meiotic germ cells.

Testicular cells are rich in PUFA (Nissen *et al*. 1978). Many fatty acids are essential for spermatogenesis, but the underlying mechanisms remain unclear. Our transcriptome results exhibit many lipid metabolism, membrane reorganizing and cell deformation processes. Rivera reported that a lack of fatty acid desaturase leads to infertility (Roqueta-Rivera *et al*. 2010). Fads2-null male mice are infertile, similar to HSL−/− mice, and cannot sustain sufficient levels of DHA, DPAn-6 or other PUFA in testis, which triggered the interruption of spermatid elongation (Roqueta-Rivera *et al*. 2010, Zadravec *et al*. 2011). We conjecture that lack of a critical unsaturated fatty acid truncates spermiogenesis in HSL−/− mice as claimed in a previous study (Casado *et al*. 2013); however, we found no significant difference in the fatty acid profiles of HSL+/+, HSL+/− and HSL−/− mouse testes (Table 1). Different gene target strains of mice were used. We used mice in which exon 1 was replaced with LacZ (Chung *et al*. 2001). However, Osuga (Casado obtained HSL−/− mice from Osuga) replaced a 12 kb DNA fragment from intron 6 with intron 7 using PstI (Osuga *et al*. 2000). We believe that the deletion of the different domains influenced the function of the gene. Especulate that the major function of HSL is not hydrolysis of glycerides to ensure homeostasis, although PUFA are indeed very important in terms of spermatogenesis. To confirm this hypothesis, we need to construct an isoform of HSL protein that exhibits only cholesterol esterase or glyceride esterase activities in the testes.

Cholesterol plays a central role in physiology, being critical in terms of the maintenance of cellular membrane structure (Salmon & Hamilton 1995); cholesterol regulates the membrane dynamics (Gondre-Lewis *et al*. 2006). Hermo reported that HSL is the only esterase that hydrolyzes cholesteryl esters in testes (Hermo *et al*. 2008), so that loss of this activity would result in cholesteryl ester accumulation and a shortage of free cholesterol, resulting in altered cholesterol homeostasis. Early studies revealed that the cholesterol level was elevated during the leptotene, zygotene and pachytene spermatocyte stages of development. The cholesterol level increases by 4–5 fold from the preleptotene to pachytene stages (Potter *et al*. 1981), but remains low at all subsequent stages of meiosis and is extremely low in mature sperm (Keber *et al*. 2013). Sperm plasma membranes contain very little cholesterol during sperm release from the seminiferous epithelium (Potter *et al*. 1981). A link between cholesterol homeostasis and fertility has been identified in patients with both hyperlipidemia and metabolic syndrome (Jaffer *et al*. 2012, Schisterman *et al*. 2014). Mutation of the cholesteryl transport protein Npc1 triggers infertility in mice (Akpovi *et al*. 2014). Injection of an inhibitor of cholesterol esterase to male mice caused an increase in the esterified cholesterol concentration in the testes, and a decrease in the weight of seminal vesicles (Bartke *et al*. 1973). One study on *Drosophila* spermatogenesis suggested that a cholesterol shortage was responsible for the defects in spermatogenesis, attributable to deficiencies in membrane temperature-sensitive remodeling (Wang & Huang 2012). HSL-deficient testes were normal in terms of fatty acid composition (Table 1), but the cholesterol concentration (Fig. 6) was abnormal. Accumulation of cholesterol esters altered cholesterol homeostasis, perhaps triggering the failure of acrosome formation or remodeling of the spermatozoon membrane.

HSL knockout creates dysfunctional gene expression and deregulation of cholesterol ester metabolism in later-stage germ cells. The transcriptome of HSL−/− sterile testes suggests that lipids play important roles in such testes. Cholesterol esters are critical HSL substrates during spermiogenesis, as confirmed by both our present results and those of a previous study. Our findings will facilitate the development of drugs for male infertility, and HSL inhibitor will be a safe male contraceptive pill. However, confirmation of the relationship between cholesterol and spermatogenesis warrants further study. It remains unclear how to reduce the substrate or increase the product in efforts to cure HSL mutation-induced male infertility. Further works will improve our understanding of the mechanism underlying the role of lipid metabolism in spermatogenesis.

## Supplementary data

This is linked to the online version of the paper at http://dx.doi.org/10.1530/REP-16-0484.

## Declaration of interest

No conflict of interest exits in the submission of this manuscript, and manuscript is approved by all authors for publication. I would like to declare on behalf of my co-authors that the work described was original research that has not been published previously, and not under consideration for publication elsewhere, in whole or in part.

## Funding

This work was sponsored by the National Natural Science Foundation of China (NSFC, No. 30870934).

## Author contribution statement

Feng Wang and Baochang Zhu conceived and designed the experiments. All authors performed the experiments. Pengcheng Jin, Feng Wang and Baochang Zhu analyzed the sequencing data. Feng Wang and Baochang Zhu wrote the manuscript.
